# Corneal Dendritic Cell Dynamics Are Associated with Clinical Factors in Type 1 Diabetes

**DOI:** 10.3390/jcm11092611

**Published:** 2022-05-06

**Authors:** Luisa H. Colorado, Lauren Beecher, Nicola Pritchard, Khaled Al Rashah, Cirous Dehghani, Anthony Russell, Rayaz A. Malik, Nathan Efron, Katie Edwards

**Affiliations:** 1 Centre for Vision and Eye Research, Queensland University of Technology, Brisbane, QLD 4069, Australia; lauren.beecher@connect.qut.edu.au (L.B.); n.pritchard@qut.edu.au (N.P.); n.efron@qut.edu.au (N.E.); katie.edwards@qut.edu.au (K.E.); 2 Security Forces Hospital Program, Riyadh 12625, Saudi Arabia; khaled.alrashah@connect.qut.edu.au; 3 School of Optometry and Vision Science, University of Canberra, Canberra, ACT 2617, Australia; cirdeh@live.com; 4 Department of Diabetes and Endocrinology, Alfred Hospital, Melbourne, VIC 3004, Australia; anthony.russell@alfred.org.au; 5 School of Public Health and Preventive Medicine, Monash University, Melbourne, VIC 3800, Australia; 6 Weill Cornell Medicine-Qatar, Doha P.O. Box 24144, Qatar; ram2045@qatar-med.cornell.edu

**Keywords:** cell dynamics, corneal immune cells, type 1 diabetes, corneal nerve plexus

## Abstract

Time-lapsed in vivo corneal confocal microscopy (IVCCM) has shown that corneal dendritic cells (DCs) migrate at approximately 1 µm/min in healthy humans. We have undertaken IVCCM of the whorl region to compare the density of rounded DCs, and DCs with (wDCs) and without (woDCs) dendrites and dynamics; trajectory (length travelled/time), displacement (distance from origin to endpoint/time) speeds and persistence ratio (displacement/trajectory) of woDCs in subjects with type 1 diabetes (T1D) (*n* = 20) and healthy controls (*n* = 10). Only the wDC density was higher (*p* = 0.02) in subjects with T1D compared to controls. There was no significant difference in cell dynamics between subjects with T1D and controls. woDC density correlated directly with HDL cholesterol (r = 0.59, *p* = 0.007) and inversely with triglycerides (r = −0.61, *p* = 0.005), whilst round-shaped cell density correlated inversely with HDL cholesterol (r = −0.54, *p* = 0.007). Displacement, trajectory, and persistency correlated significantly with eGFR (mL/min) (r = 0.74, *p* < 0.001; r = 0.48, *p* = 0.031; r = 0.58, *p* = 0.008, respectively). We show an increase in wDC density but no change in any other DC sub-type or alteration in cell dynamics in T1D. However, there were associations between DC density and lipid parameters and between DC dynamics and renal function. IVCCM provides evidence of a link between immune cell dynamics with lipid levels and renal function.

## 1. Introduction

Over the past decade, in vivo assessment of human corneal immune cells has gained importance in clinical ophthalmic research, especially since the introduction of in vivo corneal confocal microscopy (IVCCM). This technique allows the in vivo visualization of immune cells in the cornea in their natural steady-state [1,2], under stress [3,4,5], after interventional treatments in local [6,7,8] and systemic conditions [9,10], and in corneal grafts [11]. The constant traffic of dendritic cells (DCs), a subtype of immune cells, can be observed in the region of the corneal subbasal nerve plexus. Hence, imaging of this unique location not only allows the evaluation of DCs but also their interactions with the peripheral nervous system.

Corneal DCs can induce, sustain, and regulate immune response and have been categorized based on morphological appearance as round and with (mature) or without (immature) dendrites [12]. Pathological alterations have primarily been based on quantifying the density of the different DC subtypes [13]. In an experimental study in diabetic mice, corneal DC infiltration occurred, especially in obese diabetic mice and direct contact between DCs and the sub-basal nerve plexus was hypothesized to trigger nerve fiber damage [14]. However, in a separate experimental study, DCs were shown to mediate corneal nerve regeneration by releasing ciliary neurotrophic factor (CNTF), but in the cornea of Streptozotocin-T1D mice, there were reduced DCs with decreased CNTF and impaired corneal nerve innervation and regeneration [15]. We have previously shown increased corneal DC density in adolescents [16] and adults with T1D and mild diabetic neuropathy [17]. More recently, we have shown a significant correlation between corneal nerve loss and an increase in Langerhans cells (LC) density in patients with T1D, type 2 diabetes, and Latent autoimmune diabetes of adults (LADA) [18]. Changes in the density of corneal DCs may be a complex phenomenon and insufficient to capture pathology in relation to nerve damage.

More recently, time-lapse IVCCM has been used to quantify cellular dynamics (displacement, trajectory, and persistency) [19]. DC function depends on their migratory ability to specific destinations to initiate an immune response [20]. Indeed, DCs have evolved a complex and dynamic regulatory network involving different cellular and molecular interactions for the precise control of DC migration under various immunological or inflammatory conditions [21]. Distinct DC subsets have different mobilization capacities and consequently exert different immunological functions. As observed with IVCCM in steady-state conditions, round-shaped DCs, located at the corneal sub-basal nerve plexus (whorl area), move in the same direction as subbasal nerves. This cell migration pattern occurs towards the whorl and at a slower rate (0.004 µm/min) compared to nerve migration (0.05 µm/min) [22]. DCs without visible dendrites (woDCs) move rapidly in random directions (approximately 1 µm/min), and larger DCs with visible dendrites (wDCs) are more static (0.04 µm/min) than woDCs [19].

Little is known about human DC dynamics in T1D. Therefore, this study was designed to compare in vivo corneal DC density and dynamics in individuals with T1D compared to age-matched controls using “time-lapse” IVCCM. A secondary aim was to establish associations between the subsets of DCs, clinical and metabolic parameters and the severity of nerve damage.

## 2. Materials and Methods

### 2.1. Study Design

A retrospective, cross-sectional, observational, case-controlled pilot analysis was conducted. This project was approved by the Queensland University of Technology (QUT) research ethics committee (0800000167) and is in accordance with the Declaration of Helsinki. Written and informed consent of all participants was obtained prior to enrolment.

### 2.2. Participant Demographics and Lifestyle Measures

IVCCM images of 30 randomly selected participants were analyzed, including ten healthy, age-and gender-matched control subjects and 20 subjects with T1D who took part in the corneal nerve migration rate study [23] and the Longitudinal Assessment of Neuropathy in Diabetes Using Novel Ophthalmic Markers (LANDMark) study [24], respectively. Exclusion criteria included systemic disease with the potential of corneal involvement (apart from T1D for the cases), active ocular pathology, history of corneal surgery or trauma, contact lens wear, or involvement in interventional research trials. Individuals with T1D were also excluded if they had foot ulceration or infection at the time of enrolment.

The height and weight of all participants were measured, and body mass index (BMI) was calculated by dividing the weight (kg) by the square of the height of the participants (m). Smoking consumption and alcohol intake were recorded, and glycated hemoglobin A1c (HbA1c) and lipid profile were measured.

### 2.3. Assessment of Neuropathy

The neuropathy disability score (NDS) of 1–10 was used to assess signs of neuropathy. The signs assessment included quantitative sensory tests comprised of vibration perception, measured on the plantar surface of the big toe, and thermal (warm and cold) pain assessed on the dorsal surface of the foot on the hand-dominant side. Peroneal motor nerve conduction velocity (ankle to fibula head) was determined on the hand-dominant side of the participants. A diabetic neuropathy symptom score (0–4) was used to assess symptoms of neuropathy.

### 2.4. In Vivo Corneal Confocal Microscopy

IVCCM was performed in the right eye using laser scanning confocal microscopy (Heidelberg Retina Tomograph 3 Rostock Cornea Module; Heidelberg Engineering, Heidelberg, Germany) to acquire images of the cornea. With head and chin position supported by their respective rests, and through precise positioning of a moveable fixation target (slightly superior temporal in most individuals), the inferior corneal whorl and its adjacent regions, at the level of the sub-basal nerve plexus, were imaged. The vortex of the nerve plexus represented a common starting point for 13 target loops; therefore, approximately 13–26 frames of this region were captured across 15–20 min using an established method [25]. Additionally, eight non-overlapped images from the central cornea were selected for the assessment of corneal nerve parameters.

### 2.5. Image Selection

Three to five frames containing a common non-moving reference point (landmarks of nerve junctions located at the whorl), repeatedly imaged over approximately 15 min, were identified. Utilizing this landmark, the movement of surrounding cells was tracked. Two to three DCs (identifiable in all frames) with apparent movement (changed distance and shape from the landmark) were selected for analysis. When less than three cells displaying movement were evident in the selected frames, only 1–2 cells were analyzed.

### 2.6. Cell Movement Analysis

To analyze cell movement, custom-made software (TrackPoints) was used [25]. An origin, consistent with all frames for a given participant, was marked using the origin tool of the software. To minimize variability from different origin-to-reference point distances, landmarks were selected in close proximity to the chosen cells and roughly equidistant from all cells wherever applicable. Corneal nerve fibres have been previously demonstrated to exhibit minimal short-term movement (0.004 µm/min) [25]; thus, distinctive nerve junctions were utilized as “non-moving” origins. A reference “non-moving” control point was also identified and analyzed to control for any confounding movement during image processing. Approximately 3–4 time points were measured per cell at intervals of 3–5 min, and the movement parameters of 2–3 cells per participant were analyzed and averaged.

From the origin, the most distal aspects of the chosen DCs were precisely marked. The total distance travelled in each frame from time zero to time 3 or 4 by each cell (in microns) was measured and summed to obtain the total travelled length of each cell. This travelled length was then divided by the total travelled time in which movement was analyzed (in minutes) to calculate each cell’s trajectory path (µm/min).

Using the same origin, reference point and procedure, time point zero was also the starting point to calculate the direct distance from the first to last analyzed cell position in the previous step. In this part of the procedure, only two frames were analyzed (the first and last). This distance was then divided by the total time of movement to attain cell displacement (µm/min).

**Figure 1:**
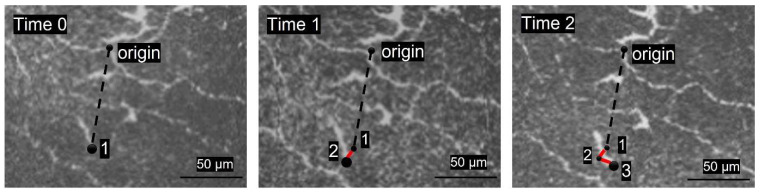
Schematic single-cell movement analysis. A nerve junction is utilized as the origin in the selected images. **Time 0** shows the most distal aspect of the chosen DC (from the origin) marked to measure cell’s trajectory path (µm/min) between the selected images. **Time 1** represents the cell distance travelled (red line) between points 1 to 2 (travelled length). **Time 2** display the total cell trajectory path (the sum of the length between point 1, 2 and 3). The cell displacement is calculated from the distance between points 1 to 3.

The persistence ratio, a representation of the tendency of a cell to adhere to a straight-line path from its first and final analyzed positions, was then calculated as the displacement divided by the trajectory path. Average trajectory speed, displacement, and persistence ratio were calculated for the analyzed cells of each participant, as shown in Figure 1.

### 2.7. Cell Counts and Density Calculation

Two frames, each containing the corneal whorl but not overlapping by more than 25%, were selected for each participant. Frames were chosen from the same consultation date as those selected for DC migration analysis. The number of cells with and without visible dendrites and round-shaped cells were counted in each frame and averaged. Cells were identified in accordance with definitions described previously: woDCs have “small, reflective cell bodies with short discernible dendrites or tapered ends”, wDCs have “reflective, slender cell bodies often with multiple long dendriform processes extending out from the main cell body”, and round-shaped cells are “rounded with uniform high reflectivity without visible dendriform protrusions and more rounded than DC bodies with no tapered ends” [26].

In participants with conglomerated clusters of round-shaped cells, rounded cell prominences at the edge of such clusters facilitated the approximation of the number of cells; if a white, hyperreflective, round mass remained after counting these prominences, one extra cell was counted, allowing recognition that more cells existed without overestimating. The number of cells was converted into cell density per millimetre squared.

### 2.8. Corneal Parameters

Semi-automated analysis was undertaken using ACCMetrics (The University of Manchester) in a masked and randomized fashion to assess corneal nerve branch density (CNBD; number/mm^2^) and corneal nerve fibre length (CNFL; mm/mm^2^) from the central cornea [27]. The corneal nerve migration rate (CNMR; µm/week) was also determined as previously described involving the TrackPoints software [23].

### 2.9. Blood Biochemistry and Metabolic Measures

HbA1c (% NGSP; mmol/mol IFCC), plasma creatinine (µmol/L), estimated glomerular filtration rate (eGFR; mL/min), total cholesterol (mmol/L), high-density lipoprotein (HDL) cholesterol (mmol/L), triglycerides (mmol/L), and low-density lipoprotein (LDL) cholesterol (mmol/L) were assessed in the T1D cohort. Any eGFR value “over 90 mL/min” was assigned a value of 91 mL/min for analysis. Urine albumin (mg/L) and urine creatinine (mmol/L) were used to calculate the albumin/creatinine ratio (mg/mmol).

### 2.10. Statistical Analysis

IBM SPSS (SPSS v 25.0; Inc., Armonk, NY, USA) was used for statistical analysis. The Shapiro–Wilk test was conducted to determine the normality of the data (α = 0.05). To compare differences between groups, parametric data were analyzed using the paired samples *t*-test, and non-parametric data were analyzed using the Mann–Whitney test. Pearson’s correlation was used to determine associations between variables. To minimize type II error, Bonferroni corrections were applied for multiple comparisons. The statistical significance level was set at *p* < 0.05.

## 3. Results

### 3.1. Participant Demographics and Lifestyle Measures

The demographic and clinical data of the participants are given in Table 1. There was no significant difference between the mean age and sex of participants with T1D and controls (*p* = 0.94 and *p* = 1.00, respectively). There were no statistically significant differences in height, weight, BMI, number of cigarettes smoked per day, and alcohol consumption (*p* > 0.08) between subjects with T1D and controls.

### 3.2. Neuropathy Measures

Diabetic neuropathy symptom scores were significantly higher in T1D compared to control subjects (*p* = 0.004) and neuropathy disability scores were significantly higher in T1D compared to controls (*p* = 0.004).

### 3.3. In Vivo Corneal Confocal Microscopy Measures

Table 2 shows the corneal nerves and dendritic cell data for the two study groups.

### 3.4. Corneal Nerve Parameters

CNBD, CNFL, and CNMR at the central cornea did not differ significantly between subjects with T1D and controls.

### 3.5. Dendritic Cell Parameters

The density of wDC at the whorl area was significantly higher in T1D compared to control subjects (13 ± 13 vs. 4 ± 4 cell/mm^2^; *p* = 0.02), but there was no significant difference for rounded cells and woDC (*p* > 0.72) between subjects with T1D and controls. There was no difference in displacement speed, trajectory path, and persistence for rounded cells, woDC and wDC between subjects with T1D and controls.

## 4. Correlations

### 4.1. Associations between Dendritic Cell Parameters and Participant Characteristics

**Figure 2:**
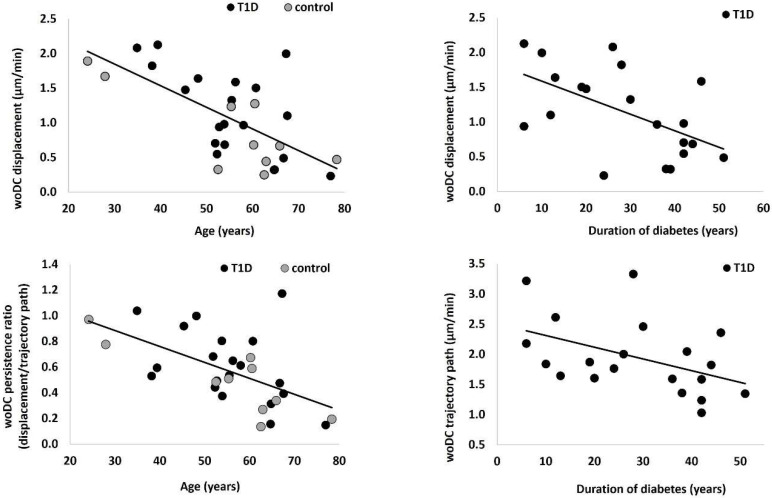
Relationship between dendritic cell dynamics and participant characteristics. woDCs; cells without visible dendrites, T1D; Type 1 diabetes.

In the entire cohort, age was inversely correlated with woDC displacement (r = −0.59, *p* = 0.001) and their tendency to continue moving in one direction, rather than randomly (r = −0.58, *p* = 0.001). In individuals with T1D, there was an inverse correlation between the duration of diabetes with woDC displacement (r = −0.50, *p* = 0.026) and the cell trajectory path (r = −0.48, *p* = 0.031), as shown in Figure 2.

### 4.2. Associations between Dendritic Cell Parameters and Metabolic Measures in Type 1 Diabetes

**Figure 3:**
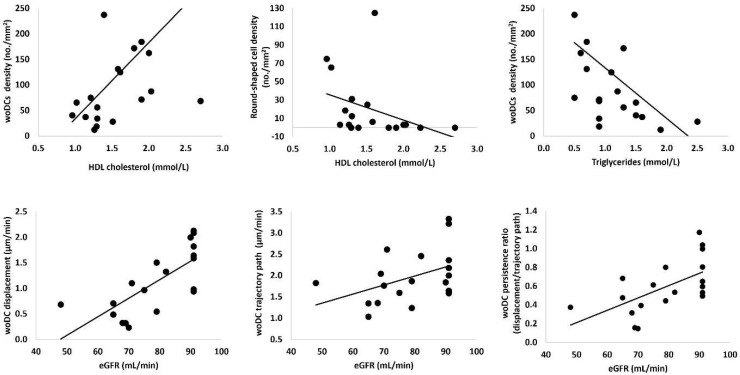
Relationship between corneal immune cell parameters and biochemistry measures in type 1 diabetes. woDCs; dendritic cells without visible dendrites, HDL; high-density lipoprotein, eCFR; estimated glomerular filtration rate.

Figure 3 shows woDC density correlated directly with HDL cholesterol (r = 0.59, *p* = 0.007) and inversely with triglycerides (r = −0.61, *p* = 0.005), whilst round-shaped cell density correlated inversely with HDL cholesterol (r = −0.54, *p* = 0.007). However, associations between cell parameters and HbA1c were not found.

The three cell dynamic parameters (displacement, trajectory, and persistency) correlated significantly with eGFR (mL/min) (r = 0.74, *p* < 0.001; r = 0.48, *p* = 0.031; r = 0.58, *p* = 0.008, respectively). However, no correlations were found between DC parameters and albumin (mg/L) measures (*p* > 0.67).

### 4.3. Associations between Dendritic Cell Parameters and Neuropathy Measures in Type 1 Diabetes

**Figure 4:**
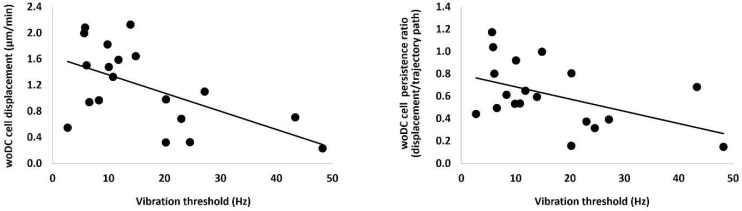
Relationship between corneal immune cell parameters and neuropathic measures in type 1 diabetes. woDCs; dendritic cells without visible dendrites.

In individuals with T1D, two cell dynamic parameters (displacement and persistency) correlated inversely with vibration threshold (Hz) (r = −0.46, *p* = 0.045 and r = −0.45, *p* = 0.046, respectively) (Figure 4). There were no other associations between DC parameters and neuropathy measures.

### 4.4. Associations between Dendritic Cell Parameters and Other IVCCM Measures

There were no associations between cell dynamics (displacement, trajectory, persistence) and DC density of any subset (round shape, wDCs, and woDCs). No associations were found between round-shaped cells and the other two cell subsets. There was a significant correlation between CNMR and cell trajectory path (µm/min) (r = 0.49, *p* = 0.016).

## 5. Discussion

This pilot study has documented DC dynamics in healthy individuals and in those with T1D and investigated factors that affect these movements. Corneal immune cell dynamics were explored retrospectively using existing data from an XY stack of IVCCM images of the nerve plexus (at the whorl) of T1D and non-diabetic participants who volunteered for previous studies undertaken at the Anterior Eye Laboratory [24,25]. The main outcome variables analyzed were DC density of cell subsets categorized by their morphological appearance (round-shaped, wDCs, and woDCs) and woDC dynamics of displacement, trajectory, and persistence. Round-shaped and wDCs were static during the 15 to 20-min observation. Previous work demonstrated that wDCs and round-shaped cells are more static compared to woDCs and corneal nerves, respectively [19,22]; therefore, only the woDC subset was analyzed for dynamic parameters.

Although there was no significant difference between DC dynamics of healthy controls and T1D participants, a strong association between cell displacement and age was observed. This association suggests that faster cell movement is indicative of healthier cell behaviour. Relatively few studies have addressed the question of migration of DCs in ageing. However, studies in humans [28] and mice [29] suggest that since the migration of DCs to lymph nodes is pivotal to the establishment of the immune response, reduced migration may contribute to age-associated immune dysfunction [28].

In support of the previous suggestion that faster movement represents healthier cell behaviour, the present study also found that all parameters of cell dynamics strongly correlated with biochemistry measures, particularly with levels of eGFR (mL/min), a measure of renal function measured in the diabetic cohort. Similar to the eye, the kidney is a non-lymphoid solid organ which suggests that the primary function of DCs is tissue homeostasis. In the kidney, following activation, DCs have to travel to the draining renal lymph node where they present antigens [30]. In the cornea, DCs are thought to also migrate to the lymph nodes through the corneal limbus under homeostatic conditions to perform antigen presentation.

Resident CD11c^+^, a subset of DCs, have been found in the cornea and kidney and exert similar roles in both organs. For example, mice studies in corneal transplants suggest that CD11c^+^ DCs isolated from donors blocked the suppressive activity of regulatory T cells in vivo [31]. Using intravitreal imaging of mouse kidney allografts, investigators also determined that CD11c^+^ DCs were potent at arresting T cells [32]. In the case of autoimmune diseases such as T1D, where the immune system is continuously activated to destroy pancreatic β-cells, a complex network of cells and mediators might respond systemically; therefore, corneal DC behaviour observed under IVCCM might represent a similar cell phenotype and behaviour in the kidney based on eGFR levels. Although a high level of albumin (above 30 mg/g) is clinically the earliest sign of kidney disease (even if the GFR is above 60), correlations between DC parameters and albumin (mg/L) were not statistically significant in the present study. This indicates that DC dynamics in the cornea might reflect kidney disease at a later stage of the condition and might not be a sensitive measure to reflect an early stage of kidney disease.

Although we did not find associations between HbA1c (a test of blood sugar levels) and DC parameters in the diabetic cohort, an interesting finding was the inverse association between round-shaped immune cells and HDL cholesterol. This negative relationship was expected as lower HDL cholesterol levels indicate poorer health, and a small number of DCs in the central cornea suggest homeostasis. Conversely, the associations of woDC with HDL cholesterol (positive) and triglycerides (negative) both suggest that higher densities of woDC are associated with better health. Depending on cell morphometry, these differences in association support the notion that different DC subsets exert different immune activity. Another reason could also be that the T1D condition exerts influence over the constantly alerted immune system. In support of this statement, this study shows that diabetic participants have a significantly higher density of wDCs at the whorl region, a sign of immune activation, compared to the healthy controls. Similar results were shown in another report on the association between DCs and diabetes mellitus [26].

Corneal nerve loss is a well-established biomarker that can predict the incidence of neuropathy with the severity of diabetic peripheral neuropathy [33]. Previous studies have also used IVCCM to report associations between DC density and the presence of neuropathy in individuals with T1D, indicating that in the earlier phases of corneal nerve damage, there is an increased number of DCs [16,17]. In support of these previous studies, we also found an increased wDC density in individuals with T1D compared to controls. Although the neuropathy status of the T1D group was not an exclusion criteria in the present study, we can assume that there was a degree of neuropathy in the T1D cohort based on the differences in the neuropathy symptom score (0–4) and neuropathy disability score (0–10) between the two groups (*p* < 0.01). Contrary, we did not find differences between the groups in the corneal nerve parameters (*p* > 0.05), possibly due to the lack of a larger cohort with established sub-groups of participants with and without diagnosed neuropathy.

In contrast, no other associations were found between DC parameters and neuropathy measures except for a weak inversed association found between DC dynamics (persistency and displacement) and vibration threshold (*p* = 0.046 and *p* = 0.045, respectively). A possible explanation for these poor associations between neuropathy measures and DC parameters might be due to how the DC were classified into subsets. A larger cohort is recommended to explore these associations in future studies.

Using IVCCM, associations between the three types of morphologically identified cells and T1D biochemistry measures have never been reported in the cornea. However, previous investigations have demonstrated morphometric changes in DCs in the presence of degenerative disorders [26,34]. Lagali et al. reported the variable density of the three subsets of DCs (round-shaped, wDCs and woDCs) present in different regions of the nerve plexus. This report suggests that the clusters of wDCs were associated with diabetes as they were proportionally less in individuals without diabetes [26].

Interestingly, the density and the trajectory path of woDCs are positively associated with CNMR. A number of corneal nerve parameters have been shown to be statistically associated with DC density [35]. However, this is the first study to demonstrate an association between DC attributes and CNMR. In support of these results, another study found no differences between CNMR in individuals with diabetes (without neuropathy) and healthy controls [25]. This corneal nerve attribute is relatively new; a limited number of studies have included CNMR in their approach, probably due to the need for customized software and a substantial number of IVCCM images and time-intensive technique to create a representative field (~1500 microns from the whorl region) to determine the measurement.

## 6. Conclusions

Corneal DC dynamics are strongly associated with biochemistry measures, notably renal function, in T1D individuals. The strong associations between renal function and corneal DC dynamics in T1D require further exploration. Other novel findings in the present study include the associations between DC trajectory path and nerve migration rate and between DC displacement and age. A larger cohort is needed to consolidate the results obtained in this study.

## Figures and Tables

**Table 1:** Clinical, metabolic and neuropathy data presented as mean ± standard deviation.

	Type 1 Diabetes (*n* = 20)	Controls (*n* = 10)	*p*
Participant Characteristics			
**Demographic and lifestyle measures**			
Age (years)	55.0 ± 11.0	55.0 ± 17.0	0.94
Sex	10/10	5/5	1.00
Height (cm)	171.6 ± 11.3	167.1 ± 7.1	0.26 *
Weight (kg)	85.0 ± 17.6	74.2 ± 10.0	0.08
BMI	28.9 ± 5.0	26.6 ± 3.4	0.21
Current smoker (Y/N)	0/10	3/17	0.53
Alcohol (units/week)	9 ± 14	4 ± 6	0.25 *
Duration of diabetes (yrs)	29 ± 14	-	-
**Neuropathy measures**			
Diabetic neuropathy symptom score (0–4)	0.9 ± 1.2	0.0 ± 0.0	<0.01 *
Neuropathy disability score (0–10)	2.9 ± 3.2	0.5 ± 0.7	<0.01 *
Peroneal nerve conduction velocity (m/s)	41.2 ± 6.1	-	
*Quantitative sensory tests*			
Cold pain threshold (°C)	6.1 ± 7.8	-	-
Warm pain threshold (°C)	48.7 ± 2.0	-	-
Vibration threshold (Hz)	16.5 ± 12.4	-	-
**Biochemistry measures**			
HbA_1C_ (%)	8.1 ± 1.0	-	-
HbA_1C_ (mmol/mol)	65.2 ± 11.1	-	-
Creatinine (mmol/L)	7.2 ± 4.3	-	-
Albumin (mg/L)	22.53 ± 55.95	-	-
Albumin-to-creatinine ratio (mg/mmol)	3.26 ± 7.07	-	-
eGFR (mL/min)	78.84 ± 12.63	-	-
Total cholesterol (mmol/L)	5.09 ± 0.81	-	-
HDL (mmol/L)	1.58 ± 0.45	-	-
LDL (mmol/L)	3.01 ± 0.64	-	-
Triglycerides (mmol/L)	1.13 ± 0.51	-	-

* Mann–Whitney Test. BMI; body mass index, HbA1C; haemoglobin A1c; eGFR; estimated glomerular filtration rate, HDL; high-density lipoprotein, LDL; low-density lipoprotein.

**Table 2:** Corneal nerves and dendritic cell data presented as mean ± standard deviation.

IVCCM Measures	Type 1 Diabetes (*n* = 20)	Controls (*n* = 10)	*p*
**Corneal nerve parameters (central cornea)**			
CNBD (no./mm^2^)	25.21 ± 15.59	34.77 ± 16.48	0.24
CNFL (mm/mm^2^)	15.32 ± 3.50	18.05 ± 3.54	0.13
CNMR (μm/week)	37.89 ± 14.92	39.24 ± 12.62	0.83
**Dendritic cell parameters (whorl region)**			
*Corneal immune cell density*			
woDCs (cell/mm^2^)	115 ± 150	85 ± 106	0.53 *
wDCs (cell/mm^2^)	13 ± 13	4 ± 4	0.02 *
Round-shaped cells (cell/mm^2^)	24 ± 37	30 ± 52	0.72 *
*Dynamics of cells without dendrites*			
Displacement speed (μm/min)	1.1 ± 0.6	0.9 ± 0.5	0.29 *
Trajectory path (μm/min)	1.9 ± 0.6	2.0 ± 1.0	0.77 *
Persistence (ratio)	0.6 ± 0.3	0.5 ± 0.3	0.29

* Mann–Whitney Test. IVCCM; in vivo corneal confocal microscopy, woDCs; dendritic cells without visible dendrites, wDCs; dendritic cells with visible dendrites, CNBD; corneal nerve branch density, CNFL; corneal nerve fiber length; CNMR; corneal nerve migration rate.

## Data Availability

The datasets used and/or analyzed during the current study are available from the corresponding author on reasonable request.
